# Positive association between nonalcoholic fatty liver disease and growth hormone deficiency in patients with nonfunctioning pituitary adenoma

**DOI:** 10.3389/fendo.2022.1057769

**Published:** 2023-01-09

**Authors:** Yoon-a Hwang, Hye Won Lee, Sang Hoon Ahn, Eun Jig Lee, Cheol Ryong Ku, Seung Up Kim

**Affiliations:** ^1^ Department of Internal Medicine, Yonsei University College of Medicine, Seoul, Republic of Korea; ^2^ Institute of Endocrine Research, Yonsei University College of Medicine, Seoul, Republic of Korea; ^3^ Pituitary Tumor Center, Severance Hospital, Seoul, Republic of Korea; ^4^ Yonsei Liver Center, Severance Hospital, Seoul, Republic of Korea

**Keywords:** nonalcoholic fatty liver disease, growth hormone, growth hormone deficiency, nonfunctioning pituitary adenoma, hepatic steatosis index

## Abstract

**Objective:**

Non-alcoholic fatty liver disease (NAFLD) is characterized by growth hormone deficiency (GHd). We investigated the association between NAFLD and GHd in patients with nonfunctioning pituitary adenomas (NFPA).

**Design and methods:**

We recruited patients with NFPA who underwent transsphenoidal adenectomy between January 2005 and December 2018. Pituitary function was determined by the insulin tolerance test, thyroid hormone assay, and gonadal hormone levels. NAFLD was defined as a hepatic steatosis index greater than 36.

**Results:**

Among 278 patients (mean age, 44.2 years; 58.6% [n=163] female), 103 (37.0%) had GHd, 139 (50.0%) had hypogonadism, and 75 (27.0%) had NAFLD. The prevalence of NAFLD was significantly higher in patients with GHd than in those without (36.9% vs. 21.1%, p=0.01). Even after adjusting for age, total cholesterol level, gonadal function, and prolactin level, patients with GHd had approximately two-fold higher prevalence of NALFD than those without GHd (adjusted odds ratio [OR]=1.85, 95% confidence interval [CI]=1.05–3.28, p=0.03). Among female patients, the prevalence of NALFD was significantly higher in those with GHd than in those without (adjusted OR=2.39, 95% CI=1.03–5.55, p=0.04); whereas, among male patients, the prevalence of NAFLD was statistically similar between those with and without GHd (p>0.05). In addition, gonadal function did not affect the prevalence of NAFLD in patients with NFPA (29.3% with eugonadism vs. 47.8% with hypogonadism, p=0.14).

**Conclusion:**

Among patients with NFPA, the prevalence of NAFLD was two-fold higher in patients with GHd than that in those without GHd. Thus, screening for NAFLD might be required in NFPA patients with GHd.

## 1 Introduction

Nonalcoholic fatty liver disease (NAFLD) is the most common cause of chronic liver disease worldwide (1). As lifestyle and dietary habits have changed, the prevalence of NAFLD has nearly doubled over the past 20 year (2). In South Korea, the prevalence of NAFLD is approximately 30% and the annual incidence of NAFLD is approximately 45 cases per 1,000 individuals (3). NAFLD is strongly associated with obesity, diabetes, hyperlipidemia, and metabolic syndrome (4–6). Furthermore, hypothyroidism, which causes metabolic impairment, is an independent risk factor for NAFLD (7, 8). In addition to hypothyroidism, several studies suggested that NAFLD is associated with hypogonadism or senescence-related hormonal insufficiency (9–13). Moreover, the association between prolactin and NAFLD has recently been suggested (14, 15).

Growth hormone (GH) is essential for maintenance of metabolic homeostasis in liver, muscle, and adipose tissues. GH regulates carbohydrate and lipid metabolism in hepatocytes. This action is mainly mediated through lipid mobilization in white adipose tissue and insulin production (16). Patients with Laron syndrome, caused by loss-of-function mutations in the GH receptor gene in humans, develop NAFLD and chronic replacement of insulin-like growth factor 1 (IGF-1) does not alleviate NAFLD status (17). In addition, liver-specific GH receptor deletion in mice results in increased hepatic insulin resistance and severe hepatic steatosis and impaired regeneration of hepatocytes, which might suggest the direct effect of GH on hepatocytes *via* the GH receptor (18).

GH deficiency (GHd) in adults clinically manifests as decreased lean body mass, muscle strength, bone mineral density, increased visceral adipose tissue, and dyslipidemia (19, 20). Several studies have investigated the association between hepatic steatosis and GHd (20–23). Steatosis severity associated with GH levels in patients with hypopituitarism (10). Liver enzymes and fatty liver improve after GH replacment in patients with GHd (22, 24). These results suggest that NAFLD may be partly attributable to GHd. However, it is not clear whether GHd is independently associated with NAFLD, because previous studies have been based on relative GHd in overweight or obese patients without the pituitary disorder (25, 26) or have small sample sizes (10, 22–24, 27–29).

In this study, we explored the associations between NAFLD and GHd in patients with nonfunctioning pituitary adenoma (NFPA) and investigated the influence of sex and gonadal function on NAFLD.

## 2 Methods

### 2.1 Study population

Patients with NFPA who underwent transsphenoidal adenectomy between January 2005 and December 2018 were recruited. The pituitary function and NAFLD were assessed before transsphenoidal adenectomy. Patients were excluded if they had any of the following (1): any diseases or were taking any drugs that can affect hormones (e.g., levothyroxine, selective estrogen receptor antagonist, glucocorticoids, etc.); (2) adrenal insufficiency or thyroid dysfunction newly diagnosed in preoperative pituitary function assessment; (3) undergone hysterectomy or unknown menstrual cycle in female patients; (4) hepatic steatosis with excessive alcohol consumption (≥210 g/week in men; ≥140 g/week in women); (5) concomitant liver diseases including viral hepatitis, autoimmune hepatitis, other causes of fatty liver disease except NAFLD; or (6) missing information. None of the patients with hypogonadism had received hormonal therapy in this study.

The study protocol was approved by the Institutional Review Board of Yonsei University Health System, Seoul, Korea (4-2022-0520) and the requirement for informed consent was waived, as this was a retrospective study.

### 2.2 Assessment of pituitary function

An insulin tolerance test was performed preoperatively to evaluate GHd and adrenal insufficiency. Regular insulin (Humulin^®^ R, Eli Lilly and Company, Indiana, USA) was injected into patients to achieve blood glucose levels of <40 mg/dL or a ≥50% decrease in glucose blood glucose level. Blood specimens were collected at 0, 30, 60, 90, and 120 min to measure GH and cortisol (30).

Blood specimens were drawn at 8 am from patients who had fasted for >8 hours. Patients on medications affecting pituitary hormones were excluded, as previously noted. Levels of free T4, thyroid-stimulating hormone, luteinizing hormone, and follicle-stimulating hormone were measured. For gonadal hormones, testosterone levels in male patients and estradiol levels in women were measured.

### 2.3 Definition of pituitary dysfunction

Patients were considered to have GHd (GHd group) if the peak GH level was below 3 ng/mL. Otherwise, they were regarded having intact GH function (GHi group) (31). Adrenal insufficiency was defined if a peak cortisol level was neither above 18mg/dL nor increased by 8 mg/dL from the baseline cortisol level (31). Central hypothyroidism was defined as (1) a thyroid-stimulating hormone level lower than or within the reference range despite low free T4 level or (2) a thyroid-stimulating hormone level lower than the reference range, free T4 level within the reference range, and thyroid-stimulating antibody titer below the cut-off value (150%) (31). In male patients, hypogonadism was considered if the testosterone level was below 250 ng/mL. Female patients were considered to have hypogonadism if they had irregular menstruation cycles or amenorrhea. Among female patients with hypogonadism, they were regarded having hypogonadotropic hypogonadism if the follicle-stimulating hormone level was less than 40 IU/L; otherwise, those were considered to be postmenopausal (32).

### 2.4 Definition of NAFLD

NAFLD was defined using a validated hepatic steatosis index (HSI) calculated as follows: HSI = 8 × alanine aminotransferase (ALT)/aspartate aminotransferase (AST) + BMI (+ 2 if diabetes yes, + 2 if female) (33). The HSI was proposed in a Korean cohort study of 10,724 subjects (5.462 subjects with NAFLD diagnosed by ultrasonography). The HSI was less than 30, and then NAFLD was excluded (negative likelihood ratio 0.2, sensitivity 93.1%). Patients were regarded as having NAFLD if HSI was greater than 36 (positive likelihood ratio 6.1, specificity 92.4%) (33). The AUC of the HSI was 0.81 and an acceptable accuracy among a Korean population (33, 34). The primary outcome was the association between NAFLD and GH status.

### 2.5 Statistical analysis

Continuous variables are expressed as means and standard deviations and categorical variables are presented as numbers and percentages. Mean values between groups were compared using the Mann-Whitney test. The proportions between the groups were compared using the chi-square test. Multivariable logistic regression analysis was applied to determine the independent association between GHd and NAFLD after adjusting for age in model 1, age and cholesterol level in model 2, and age, cholesterol level, hypogonadism, and prolactin level in model 3. Statistical analyses were performed using SPSS version 26.0 for Windows (IBM Corp., Armonk, NY, USA). For all calculations, a p value <0.05 was considered statistically significant.

## 3 Results

### 3.1 Baseline characteristics of the study population

**Figure 1:**
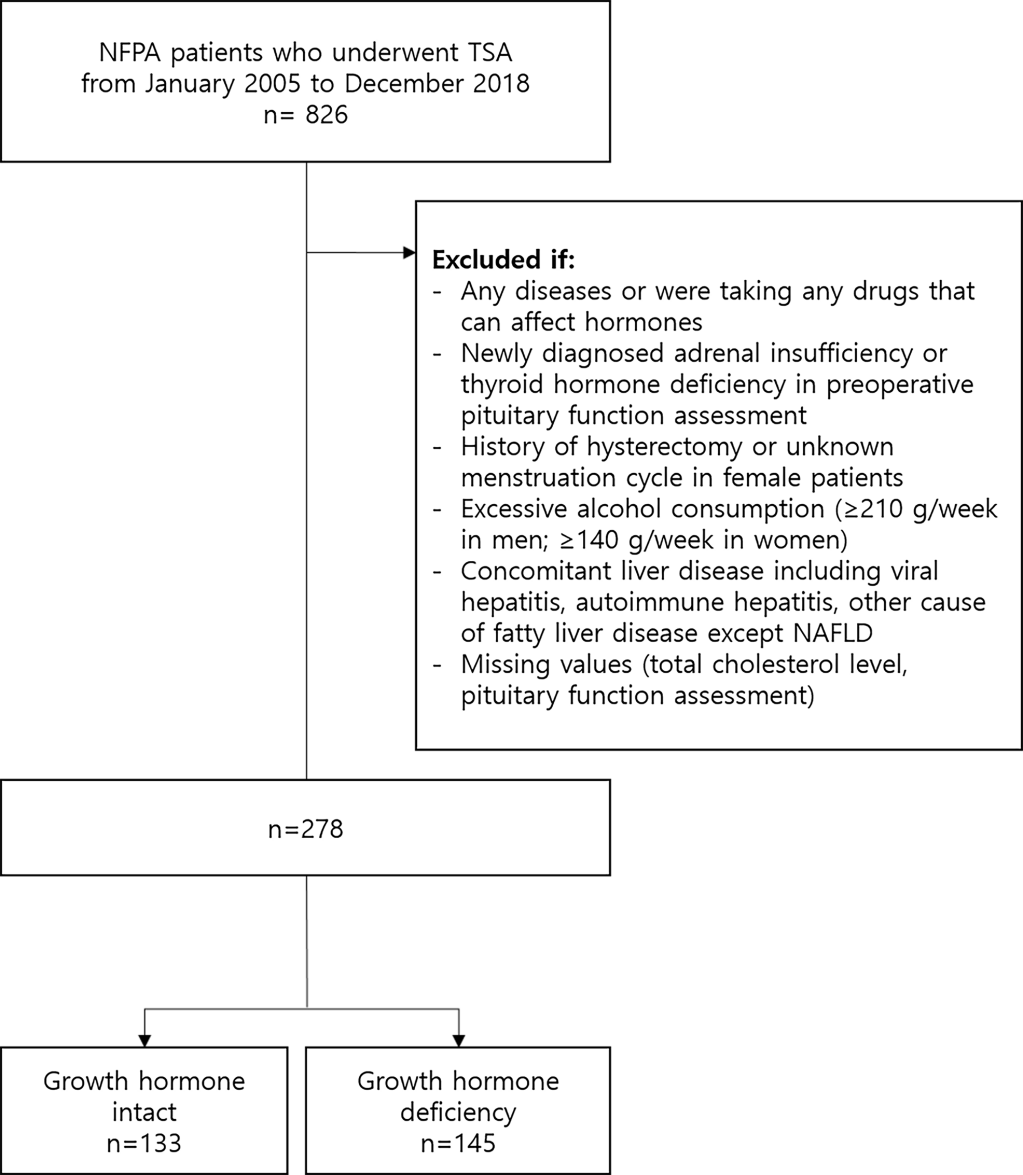
Flow diagram of subject inclusion and exclusion. NFPA, nonfunctioning pituitary adenoma; TSA, transsphenoidal adenomectomy; NAFLD, nonalcoholic fatty liver disease.

After excluding 548 patients according to our exclusion criteria, 278 patients (mean 44.2 years; 58.6% [n=163] female) were selected for the statistical analysis (**Figure 1**) The mean BMI and total cholesterol level were 23.8 kg/m^2^ and 190 mg/dL, respectively. Eighteen (6.5%) patients had diabetes. 103 (37.0%) patients had GHd and 139 (50.0%) patients had hypogonadism. 46 (16.5%) patients had isolated GHd and 82 (29.5%) patients had only hypogonadism. 57 (20.5%) patients had both. The mean prolactin level was 29.4 ng/mL. 75 (27.0%) patients had NAFLD.

### 3.2 Comparison between patients with and without GHd

The GHd group had a significantly older age (mean 46.5 vs. 42.9 years, p=0.01), higher BMI (mean 24.8 vs. 23.4 kg/m^2^, p=0.001), and higher total cholesterol level (mean 203 vs. 189 mg/dL, p=0.001) than the GHi group (**Table 1**). The prevalence of NAFLD was significantly higher in the GHd group than that in the GHi group (36.9% vs. 21.1%, p=0.01).

**Table 1:** Basic characteristics of population.

Variables	Total (n=278)			Male (n=115)			Female (n=163)		
	GHi	GHd	p-value	GHi	GHd	p-value	GHi	GHd	p-value
	(n=175, 63%)	(n=103, 37%)		(n=58, 50%)	(n=57, 50%)		(n=117, 72%)	(n=46, 28%)	
Age (yrs)	42.9 ± 12.5	46.5 ± 12.1	0.01	50.1 ± 12.1	50.3 ± 11.5	0.91	39.3 ± 11.2	41.7 ± 11.1	0.13
Body mass index (kg/m^2^)	23.4 ± 3.3	24.8 ± 3.3	0.001	24.6 ± 2.4	25.3 ± 2.7	0.15	22.8 ± 3.5	24.1 ± 3.9	0.04
Diabetes	10 (5.7)	8 (7.8)	0.62	7 (12.1)	6 (10.5)	0.99	3 (2.6)	2 (4.3)	0.62
Total cholesterol (mg/dL)	190 ± 34	204 ± 40	0.001	185 ± 34	203 ± 42	0.01	192 ± 33	204 ± 39	0.04
Hypogonadism	82 (46.9)	57 (55.3)	0.21	5 (8.6)	18 (31.6)	0.002	77 (65.8)	39 (84.8)	0.02
Hypogonadotropic hypogonadism	–	–	–	–	–	–	64 (54.7)	35 (76.1)	0.01
Prolactin (ng/ml)	31.3 ± 29.2	26.1 ± 25.6	0.124	11.2 ± 8.2	14.0 ± 9.8	0.09	41.1 ± 30.7	41.7 ± 31.0	0.91
NAFLD	37 (21.1)	38 (36.9)	0.01	16 (27.6)	22 (38.6)	0.24	21 (17.9)	16 (34.8)	0.04

Variables are expressed as mean ± SD or n (%).

GHi, intact growth hormone function; GHd, growth hormone deficiency; NAFLD, nonalcoholic fatty liver disease.

GHd was defined as peak growth hormone < 3 ng/mL in insulin tolerance test and NAFLD was defined as hepatic steatosis index greater than or equal to 36.

### 3.3 Comparison between patients with and without GHd according to sex

The baseline characteristics of the patients with and without GHd were compared according to sex (**Table 1**). In 115 (41.4%) male patients, the mean age and BMI were 50.2 years and 24.9 kg/m^2^, respectively. Thirteen (11.3%) patients had diabetes. 57 (49.6%) patients had GHd, and 23 (20.0%) patients had hypogonadism. The number of patients with NALFD was 38 (33.0%). Total cholesterol levels were significantly higher in the GHd group than in the GHi group (203 mg/dL vs. 185 mg/dL, p=0.002).

In 163 (58.6%) female patients, the mean age and BMI were 40.0 years and 23.1 kg/m^2^, respectively. Five (3.1%) female patients had diabetes. The number of patients with GHd or patients with hypogonadism was 46 (28.3%) and 116 (71.2%), respectively. 16 (34.8%) female patients had NALFD. Female patients with hypogonadism were further subdivided into postmenopausal and hypogonadotropic hypogonadism groups. Among female patients with hypogonadism, 99 (85.3%) had hypogonadotropic hypogonadism and 17 (14.7%) were postmenopausal. Similar to the male group, the total cholesterol level was significantly higher in the GHd group than in the GHi group (204 vs. 192 mg/dL, p=0.04).

### 3.4 Association between growth hormone status and NAFLD

**Figure 2:**
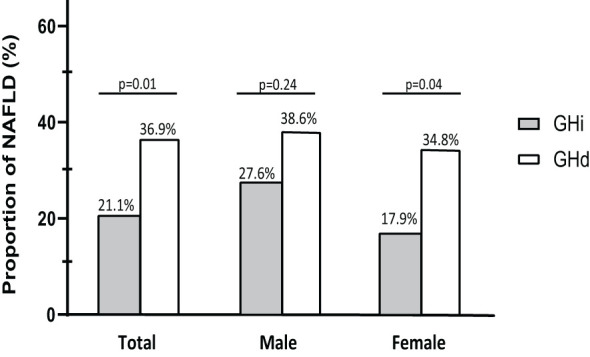
Prevalence of NAFLD according to growth hormone status stratified by gender. NAFLD, nonalcoholic fatty liver disease; GHi, intact growth hormone function; GHd, growth hormone deficiency.

The association between growth hormone status and NALFD was assessed (**Figure 2** and **Table 2**). The prevalence of NAFLD was significantly higher in the GHd group than in the GHi group (36.9% vs. 21.1%, p=0.01). GHd was significantly associated with NAFLD (unadjusted odds ratio [OR]=2.19, 95% confidence interval [CI] 1.27-3.74, p=0.01). In a fully adjusted model (Model 3), the GHd group had a 1.85-fold increased risk of NAFLD compared with the GHi group (adjusted OR=1.85, 95% CI 1.05-3.28, p=0.03).

**Table 2:** Unadjusted and adjusted odds ratio of NAFLD in regard to growth hormone status in patients with NFPA.

	Total		Female	
	Odds ratio (95% CI)	p-value	Odds ratio (95% CI)	p-value
Unadjusted	2.18 (1.27, 3.74)	0.01	2.50 (1.18, 5.31)	0.02
Model 1	2.04 (1.18, 3.53)	0.01	2.27 (1.02, 5.03)	0.04
Model 2	2.01 (1.16, 3.50)	0.01	2.11 (0.94, 4.74)	0.07
Model 3	1.85 (1.05, 3.28)	0.03	2.39 (1.03, 5.55)	0.04

NAFLD, nonalcoholic fatty liver disease; CI, confidence interval; NFPA, nonfunctioning pituitary adenoma.

Model 1: adjusted by age.

Model 2: adjusted by age, preoperative total cholesterol.

Model 3: adjusted by age, preoperative total cholesterol, hypogonadism, and prolactin level.

### 3.5 Association between sex and NAFLD

The association between sex and NALFD was also assessed (**Figure 2** and **Table 2**). The prevalence of NFALD was statistically similar between the GHd and GHi groups among the male patients (38.6% vs. 27.6%, p=0.24). In contrast, among the female patients, the GHd group had a significantly higher prevalence of NAFLD than the GHi group (34.8% vs. 17.9%, p=0.04). In a fully adjusted model (Model 3), the GHd group had the increased risk of NAFLD compared with the GHi group (adjusted OR=2.39, 95% CI 1.03-5.55, p=0.04).

### 3.6 Association between gonadal dysfunction and NAFLD

The prevalence of NAFLD did not differ according to gonadal function (28.1% in eugonadism vs. 25.9% in hypogonadism; p=0.79). When divided according to sex, similar results were maintained (29.3% in eugonadism vs. 47.8% in hypogonadism in male patients, p=0.14; 25.5% in eugonadism, vs. 29.4% in postmenopausal, vs. 20.2% in hypogonadotropic hypogonadism, p=0.57) (**Supplement Table 1**).

## 4 Discussion

To the best of our knowledge, this is the first study to analyze the association between GHd and NAFLD, excluding the effects of other hormones, including thyroid hormones. We demonstrated that NFPA patients with GHd showed a 2.18 times increased risk of NAFLD compared with GHi patients. The risk of NAFLD remained higher in the GHd group after adjusting for age, total cholesterol level, hypogonadism, and the prolactin level (OR=1.85). No statistically significant difference was observed in the prevalence of NFALD between the GHd and GHi groups among male patients, whereas the risk of NAFLD was 2.5 times higher in the GHd group than in the GHi group among female patients. The increased risk of NFALD persists after adjusting for age, total cholesterol level, hypogonadism, and the prolactin level (OR=2.39) in female patients. Hypogonadism was not significantly associated with NAFLD in this study.

Our study has several implications. First, we demonstrated an independent association between GHd and NAFLD. Although the causal relationship between the two factors could not be assessed in a retrospective cross-sectional study, our main finding is supported by previous basic and clinical research (10, 17, 21–23, 35–39). GH indirectly affects hepatic steatosis by changing body composition. GHd in adulthood increases fat mass, predominantly in the abdominal compartment (40) and visceral fat is significantly associated with hepatic steatosis (41, 42). In *in vivo* experiments using hepatocyte-specific GHR knockdown (aHepGHRkd) mice, hepatic steatosis rapidly developed independently of systemic insulin sensitivity and lipolysis (37, 43). In addition, in *in vitro* and *in vivo* experiments, GHd prevented the activation of signal transducer and activator of transcription-5 (STAT5), causing an increase in liver lipid uptake and promoting the development of NAFLD (21, 44).

Second, although, to date, few studies have explored the association between GHd and NAFLD, the results have been conflicting. Hong et al. reported that the severity of hepatic steatosis in 34 male patients with hypopituitarism was associated with GHd after adjusting for the BMI effect (10). Nishizawa et al. also demonstrated a higher rate of NAFLD in GHd subjects when diagnosed with ultrasonography (22). On the other hand, Meienberg et al. reported that there was no difference in intra-hepatocellular lipid components measured using MRI in 22 adults with GHd compared with healthy controls after adjusting for age, race, height, weight, and sex (29). Another study conducted by Gardner et al. showed no difference in the prevalence of NAFLD between adult patients with GHd and controls (36). The average BMI of the study by Gardner et al. was higher than those of the studies by Hong et al. or Nishizawa at el (27.8 kg/m^2^ vs. 25.2 kg/m^2^ and 25.0 kg/m^2^). The discrepant results regarding the association between GHd and NAFLD prevalence might suggest that the effects of GHd on liver fat may vary according to ethnicity (29). In addition, this discrepancy may be because the effect on hypothyroidism, which has recently been shown to be related to NAFLD (38, 45, 46), was not excluded in other studies. In contrast, we proved an association between GHd and NAFLD after excluding the confounding effect of other hormones on NAFLD. Patients with hypothyroidism or adrenal insufficiency were excluded as previously stated. Additionally, gonadal function and the prolactin level were incorporated into multiple logistic regression model to minimize the effect of hypogonadism on NAFLD.

Third, in our study, an increased risk of NAFLD in the GHd group was maintained only in female patients, not in male. Although previous studies reported that baseline GH levels were not different between sexes (47), it is known that estrogen stimulates GH secretion while inhibiting insulin-like growth factor 1 production in the liver, which enhances GH secretion (48, 49). However, in our study, the risk of NAFLD in female patients was still increased in the GHd group after adjusting for hypogonadism. Although no study has compared metabolic complication between male and female patients with GHd, it is known that female patients with GHd require a higher dose of recombinant GH than their male counterparts (50). In addition, Franco et al., showed that the change in visceral fat mass in postmenopausal women after GH treatment were less than that in age- and BMI-matched men (51). The observed difference in the risk of NAFLD in this study, along with previous studies, might suggest the sex dimorphism of GH action in the liver that was not solely caused by estrogen.

Fourth, in epidemiological studies, the prevalence of NAFLD in women of reproductive age is lower than that in their male counterparts; however, postmenopausal women have a comparable prevalence of NAFLD with men (52). The prevalence of NAFLD increases not only in postmenopausal women (53) but also in those with iatrogenic menopause (54), suggesting a protective effect of estrogen. In men, low testosterone levels are known to be associated with NAFLD (11–13). In contrast, our results showed no association between hypogonadism and NAFLD prevalence. Rather than hypogonadism having no effect on NAFLD, it is possible that most female patients in this study were in premenopausal age and the duration of exposure to hypogonadism might not have been long enough to induce fatty liver. In the case of male patients, the inconsistent results may be due to the different cut-off values of testosterone for male hypogonadism were set for each study (11–13).

Despite its several strengths and clinical implications, our study has several limitations. First, although we used well-validated surrogate for the diagnosis of NAFLD (3, 33, 34), liver imaging and histological information for assessing fatty liver were not available. Second, due to the cross-sectional nature of our study design, we could not assess the longitudinal dynamic association between the development of hepatic steatosis and GHd. Third, the duration of exposure to GHd was unclear. The relation between the exposure period, severity of GHd, and the risk of NAFLD may have been biased. Lastly, we could not examine the effects of anthropic or muscle mass, which are significant risk factors for NAFLD.

In conclusion, among patients with NFPA, the prevalence of NAFLD was two-fold higher in patients with GHd than in those without GHd. Thus, screening for NAFLD might be required in patients with NFPA, if GHd is present. However, further studies are needed to evaluate the dynamic association between GHd levels and NAFLD incidence.

## Data availability statement

The raw data supporting the conclusions of this article will be made available by the authors, without undue reservation.

## Ethics statement

The studies involving human participants were reviewed and approved by Institutional Review Board of Yonsei University Health System. Written informed consent for participation was not required for this study in accordance with the national legislation and the institutional requirements.

## Author contributions

Conceptualization and design: CK and SK; writing original draft: Y-AH and HL; writing review, and/or revision of the manuscript: Y-AH, HL, SA, EL, CK, and SK; funding acquisition: CK and SK. All authors contributed to the article and approved the submitted version.
