# Supplementary material for: Positive association between nonalcoholic fatty liver disease and growth hormone deficiency in patients with nonfunctioning pituitary adenoma
Source: Front Endocrinol (Lausanne). 2023 Jan 9;13:1057769. doi: 10.3389/fendo.2022.1057769 (PMC9868829; doi:10.3389/fendo.2022.1057769)
Supplement: Supplementary file 1 [file Table_1.docx]

Supplement Table 1. Association between NAFLD and hypogonadism.

|  | | | Total | Without NAFLD | NAFLD | p-value |
| --- | --- | --- | --- | --- | --- | --- |
| Total | | Eugonadism | 139 | 100 (71.9%) | 39 (28.1%) | 0.79 |
|  |  | Hypogonadism | 139 | 103 (74.1%) | 36 (25.9%) |  |
| Male | | Eugonadism | 92 | 65 (70.7%) | 27 (29.3%) | 0.14 |
|  |  | Hypogonadism | 23 | 12 (52.2%) | 11 (47.8%) |  |
| Female | | Eugonadism | 47 | 35 (74.5%) | 12 (25.5%) | 0.57 |
|  |  | Postmenopause | 17 | 12 (70.6%) | 5 (29.4%) |  |
|  |  | Hypogonadotropic hypogonadism | 99 | 79 (79.8%) | 20 (20.2%) |  |
|  | NAFLD, nonalcoholic fatty liver disease, was defined as hepatic steatosis index greater than or equal to 36. | | | | | |
